# How to train a neuron

**DOI:** 10.7554/eLife.00491

**Published:** 2013-01-22

**Authors:** Rui P Costa, Alanna J Watt, P Jesper Sjöström

**Affiliations:** 1 **Rui P Costa** is at the Institute for Adaptive and Neural Computation, University of Edinburgh, United Kingdomrui.costa@ed.ac.uk; 2 **Alanna J Watt** is at the Department of Biology, McGill University, Montréal, Canadaalanna.watt@mcgill.ca; 3 **P Jesper Sjöström** is at the Department of Neurology and Neurosurgery, McGill University Health Centre, Montréal, Canadajesper.sjostrom@mcgill.ca

**Keywords:** synaptic plasticity, STDP, visual cortex, circuits, in vivo, spiking patterns, Rat

## Abstract

A cellular learning rule known as spike-timing-dependent plasticity can form, reshape and erase the response preferences of visual cortex neurons.

**Related research article** Pawlak V, Greenberg D, Sprekeler H, Gerstner W, Kerr J. 2013. Changing the responses of cortical neurons from sub- to suprathreshold using single spikes in vivo. *eLife* **2**:e00012. doi: 10.7554/eLife.00012

**Image** Visual cortex neurons alter their responses using spike-timing-dependent plasticity in vivo

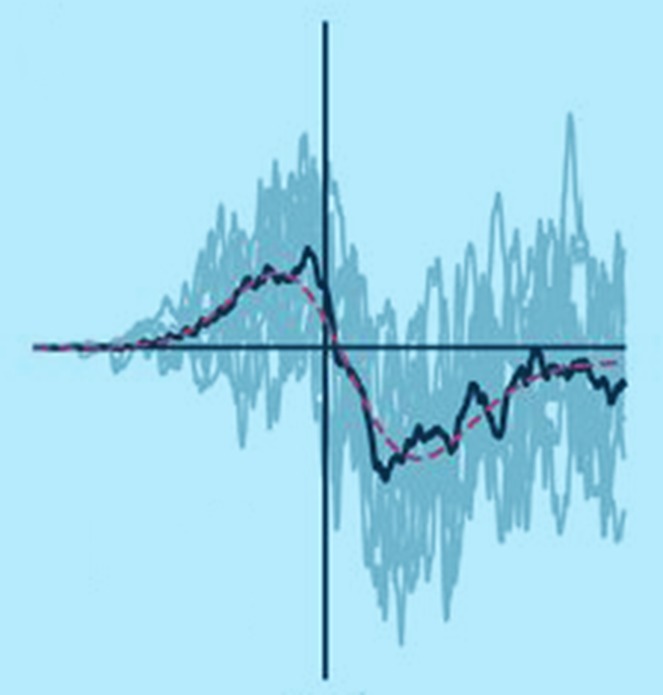

How does the brain wire itself up? This is an important question because the patterning of synaptic connections between neurons determines brain functioning. Changes in synaptic connections are governed by cellular learning rules. The synaptic plasticity that enables these changes to take place is at a maximum in the developing brain, which uses sensory input to refine patterns of connectivity as the animal learns about the outside world. Indeed, during a limited time-window known as the critical period, sensory input is essential for establishing proper connectivity. When the critical period is over, this potential for plasticity—and for learning—is diminished.

Neuroscientists have characterized several cellular learning rules in vitro, but it is unclear which, if any, of these have functional relevance in vivo. One such rule is spike-timing-dependent plasticity (STDP), whereby changes in the strength of neuronal connections depend acutely on the precise timing of spikes, or action potentials, in connected cells (Markram et al., 1997). Imagine two connected neurons, ‘A’ and ‘B’ (Figure 1). If cell ‘A’ spikes a few milliseconds before cell ‘B’, the connection between the two will be strengthened, whereas if cell ‘B’ spikes before cell ‘A’, the connection will be weakened. Although STDP is attractive as a cellular learning rule (Markram et al., 2012), its biological relevance has been called into question because most STDP experiments have been carried out in dissected brain tissue (Frégnac et al., 2010; Lisman and Spruston, 2010).

**Figure 1.**
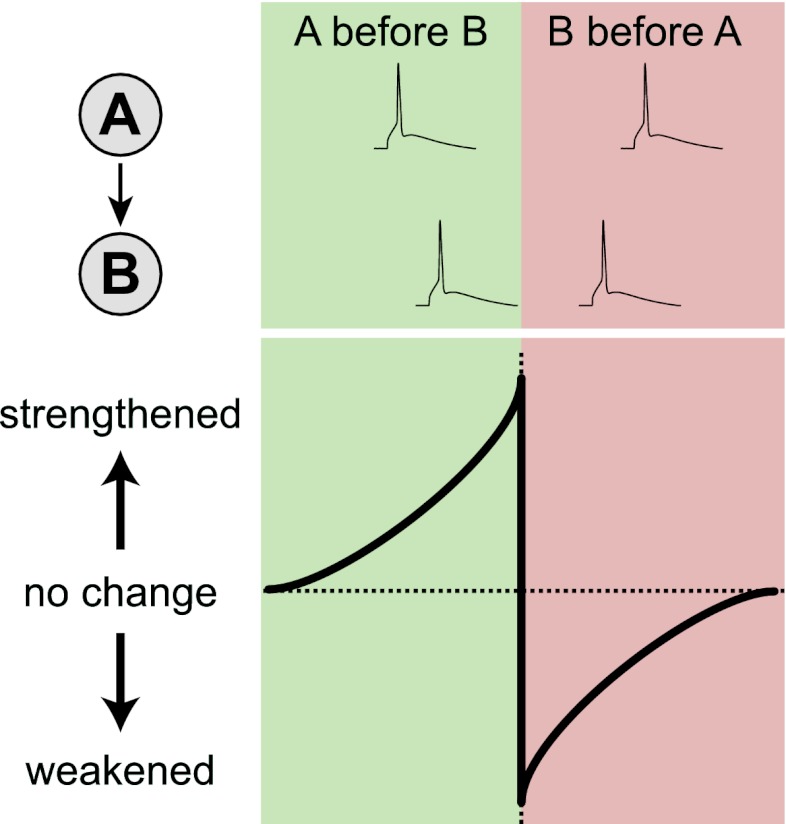
In STDP, neuronal connections change strength depending on the relative timing of spikes. The lower figure shows how the strength of a connection between cell A and cell B changes as a function of the time difference between the spikes. Cell A consistently spiking before cell B (green region) strengthens the A→B connection, whereas cell B spiking before cell A (red region) weakens the connection. In dissected brain tissue, these changes occur over a time scale of approximately 50 milliseconds (Markram et al., 2012). However, Pawlak and colleagues found that they occur over a time scale of approximately 250 milliseconds in the intact brain.

So does the brain use STDP? Now, writing in *eLife*, Verena Pawlak and Jason Kerr of the Max Planck Institute for Biological Cybernetics, and their colleagues report on heroic experiments in rats that take a key step towards answering this question (Pawlak et al., 2013). They performed technically challenging in vivo whole-cell recordings of putative pyramidal neurons in layer 2/3 of the visual cortex, during the critical period when the circuitry is most plastic. Neurons in primary visual cortex are tuned to specific stimuli: a neuron may, for example, spike preferentially in response to a specific visual stimulus in a certain part of the visual field. This neuron will, in addition, produce non-spiking responses to stimuli presented in other regions of visual space, referred to here as its sub-threshold receptive field.

To assess the importance of STDP in the visual cortex, Pawlak, Kerr and co-workers used a visual stimulus (a bar presented for half a second) to evoke a response in a neuron, and paired this repeatedly with a brief injection of current to elicit a spike (Figure 2A). By varying the relative timing of these two inputs, they were able to conduct three key experiments that demonstrate cellular learning, re-learning, and unlearning.

**Figure 2.**
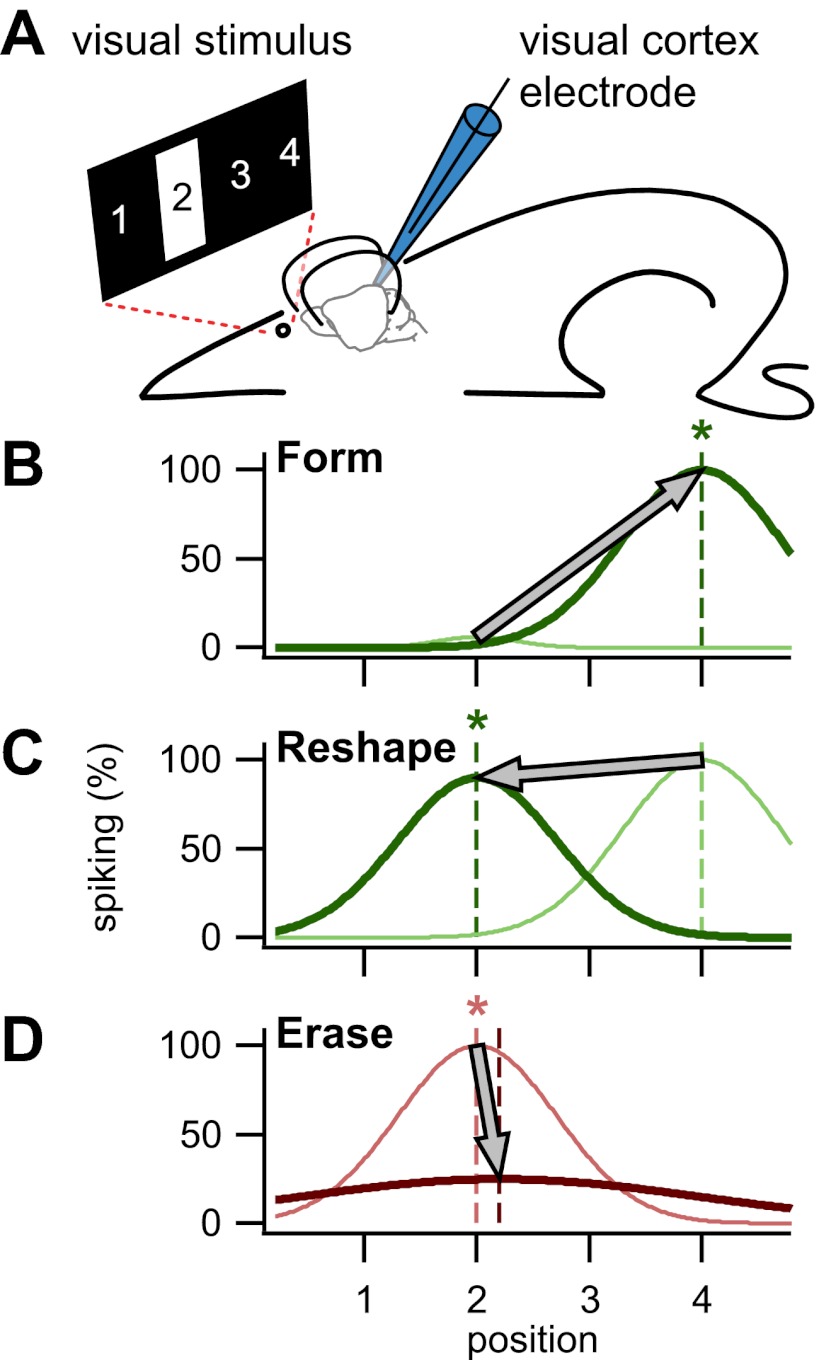
Using STDP to train visual cortex neurons in rats. (**A**) In the setup used by Pawlak and colleagues, a bar was presented in one of four positions in the neuronal receptive field, position 2 in this case. A patch electrode recorded the activity of an individual neuron, and was also used to elicit single spikes by a brief injection of current. (**B**) By repeatedly eliciting a spike milliseconds after presentation of a visual stimulus in position 4, the neuron was trained to respond to that stimulus: the dark green line is the newly formed tuning curve; the pale green line is before training. (**C**) It was also possible to reshape an existing tuning curve by pairing the spike with the visual stimulus in a non-preferred position (in this case position 2). (**D**) By eliciting the spike milliseconds before a preferred visual stimulus, tuning was erased. Asterisks denote the trained position, while colors correspond to timings as in Figure 1.

First, they showed that a naive untuned neuron—that is, one that responds with little or no selectivity to a stimulus at any location in its receptive field—could be trained to spike whenever the visual stimulus occupied a specific position chosen by the experimenter (Figure 2B). To do this, they presented the stimulus in the desired position, and then a few milliseconds later, injected current through the recording electrode to elicit a spike. Repeatedly pairing the visual evoked response with the spike, in that order, strengthened the association between the two in accordance with STDP. Second, they showed that a neuron tuned to a particular location could be re-trained to spike when the visual stimulus was in a different position (Figure 2C) by repeating what they did in the first set of experiments (that is, by injecting current a few milliseconds after the visual stimulus was presented). Again this was in agreement with STDP. Last, they demonstrated that reversing the order of repeated spike–response pairings (that is, by triggering a spike and then presenting the visual stimulus) erased the tuning (Figure 2D), also in agreement with STDP.

When they examined the impact of these pairing events on the sub-threshold visual responses of the neurons, Pawlak and colleagues found that responses preceding the spike were strengthened while those following it were weakened. This biphasic change is consistent with STDP (Figure 1), and explains why the temporal order of response-spike pairings brings about either learning or unlearning (Figure 2). Surprisingly, however, the biphasic changes occurred over a time scale five-fold longer than that anticipated from typical STDP studies in vitro (Markram et al., 2012). Using a computer model, Pawlak and co-workers showed that this temporal rescaling could result from noise in the spike timing of inputs. Such noise is to be expected in the intact brain, where there is always ongoing activity, but not in dissected brain tissue, which is relatively inactive.

Although a previous study has already shown that sub-threshold, non-spiking responses retune in visual cortical neurons in accordance with STDP (Meliza and Dan, 2006), Pawlak and colleagues go further by showing that STDP can control neuronal spiking output. This is important, because spikes are needed to convey stimulus feature information to other neurons. Their work is also reminiscent of a classical study of cellular learning in cat visual cortex, where responses were altered by pairing visual input with direct visual cortex stimulation or inhibition (Frégnac et al., 1988). However, Pawlak and colleagues’ work reveals the millisecond timing requirements for cellular learning, suggesting a physiological relevance for STDP.

It is important to note that these findings were obtained in anaesthetized animals, and remain to be confirmed in the awake state. Indeed, factors such as attention are likely to influence cellular learning processes (Markram et al., 2012). Although the current results show that STDP can support cellular learning, they do not reveal which synapses were altered. Finally, the evoked training spikes could be regarded as relatively artificial stimuli, which has been a criticism of STDP protocols in the past (Lisman and Spruston, 2010). It will be interesting to see whether similar results can be obtained using more natural spiking patterns. Despite these limitations, the elegant work of Pawlak, Kerr and colleagues provides some of the strongest evidence to date that STDP may underlie cellular learning in the intact brain.

## References

[bib1] Frégnac Y Pananceau M René A Huguet N Marre O Levy M 2010 A re-examination of Hebbian-covariance rules and spike timing-dependent plasticity in cat visual cortex in vivo. Front Synaptic Neurosci 2:147 21423533 10.3389/fnsyn.2010.00147 PMC3059677

[bib2] Frégnac Y Shulz D Thorpe S Bienenstock E 1988 A cellular analogue of visual cortical plasticity. Nature 333:367–70 doi: 10.1038/333367a0 3374571

[bib3] Lisman J Spruston N 2010 Questions about STDP as a general model of synaptic plasticity. Front Synaptic Neurosci 3:5 10.3389/fnsyn.2010.00140 PMC3059684 21423526

[bib4] Markram H Gerstner W Sjöström PJ, editors. 2012 Spike-timing-dependent plasticity: a comprehensive overview. Lausanne, Switzerland: Frontiers Media SA doi: 10.3389/978-2-88919-043-0 PMC3395004 22807913

[bib5] Markram H Lübke J Frotscher M Sakmann B 1997 Regulation of synaptic efficacy by coincidence of postsynaptic APs and EPSPs. Science 275:213–5 8985014 10.1126/science.275.5297.213

[bib6] Meliza CD Dan Y 2006 Receptive-field modification in rat visual cortex induced by paired visual stimulation and single-cell spiking. Neuron 49:183–9 doi: 10.1016/j.neuron.2005.12.009 16423693

[bib7] Pawlak V Greenberg D Sprekeler H Gerstner W Kerr J 2013 Changing the responses of cortical neurons from sub- to suprathreshold using single spikes in vivo. eLife 2:e00012 doi: 10.7554/eLife.00012 PMC3552422 23359858

